# Block and Boost DNA Transfer: Opposite Roles of OmpA in Natural and Artificial Transformation of *Escherichia coli*

**DOI:** 10.1371/journal.pone.0059019

**Published:** 2013-03-22

**Authors:** Dongchang Sun, Bing Wang, Lihong Zhu, Mengyao Chen, Linlin Zhan

**Affiliations:** 1 State Key Laboratory Breeding Base for Zhejiang Sustainable Pest and Disease Control, Zhejiang Academy of Agricultural Sciences, Hangzhou, P. R. China; 2 Institute of Plant Protection and Microbiology, Zhejiang Academy of Agricultural Sciences, Hangzhou, P. R. China; University of Manchester, United Kingdom

## Abstract

Our previous work established that DNA is naturally transferable on agar plates through a new transformation system which is regulated by the stationary phase master regulator RpoS in *Escherichia coli*. In this transformation system, neither additional Ca^2+^ nor heat shock is required. Instead, transformation is stimulated by agar. The membrane protein OmpA, a gated pore permeable to ions and larger solutes, serves as a receptor for DNA transfer during bacteriophage infection and conjugation. However, it remains unknown how DNA transfers across membranes and whether OmpA is involved in transformation of *E. coli*. Here, we explored potential roles of OmpA in natural and chemical transformation of *E. coli*. We observed that *ompA* inactivation significantly improved natural transformation on agar plates, indicating that OmpA blocks DNA transfer. Transformation promotion by *ompA* inactivation also occurred on soft plates, indicating that OmpA blocks DNA transfer independent of agar. By contrast, compared with the wild-type strain, chemical transformation of the *ompA* mutant was lower, indicating that OmpA has a role in DNA transfer. Inactivation of *ompA* also reduced chemical transformation in solution containing less Ca^2+^ or with a shortened time for heat shock, suggesting that the promotion effect of OmpA on DNA transfer does not solely rely on Ca^2+^ or heat shock. We conclude that OmpA plays opposite roles in natural and chemical transformation: it blocks DNA uptake on agar plates but promotes DNA transfer in the liquid Ca^2+^ solution. Considering that no single factor was identified to reverse the function of OmpA, we propose that multiple factors may cooperate in the functional reversal of OmpA during natural and artificial transformation of *E. coli*. Finally, we observed that *ompA* transcription was not affected by the expression of RpoS, excluding the possibility that RpoS regulates DNA transfer by suppressing *ompA* transcription.

## Introduction

Horizontal gene transfer (HGT) provides bacteria with new genetic traits to better survive in the changing environment [1]. Consequently, some pathogens improve their virulence and resistance to better combat with the host immune system [2]. Three forms of HGT exist in nature: transformation, conjugation and bacteriophage infection [3]. In both conjugation and bacteriophage infection, transferring DNA is coated with proteins which help DNA enter the recipient cells. By contrast, naked DNA spontaneously enters a competent bacterium during transformation. Conjugation and bacteriophage infection have been well documented in *Escherichia coli* for a long time [4], [5], but this species is commonly known as a non-naturally transformable bacterium because it was transformable only with artificial treatments (e.g. heat shock and a high concentration of divalent cations) [6]–[9]. Recently, transformation of *E. coli* on agar plates without these artificial treatments has been repeatedly reported by several independent groups [10]–[13].

The bacterial membrane is a protein-containing lipid bilayer, which separates the interior of the cell from the outside environment. In natural transformation, exogenous DNA needs to overcome the membrane barrier [14]. In many naturally transformable bacteria (e.g. *Bacillus subtilis* and *Haemophilus influenzae*), a group of membrane and periplasmic proteins mediate the transfer of DNA across the bacterial membrane barrier [3]. Genomic analysis revealed that *E. coli* possesses a set of genes potentially encoding these proteins for DNA uptake [15]. Transcriptomic analysis indicated that the activation of the DNA uptake regulator Sxy, whose homologs control the development of competence for transformation in *H. influenzae* (*Pasteurellaceae*) and *Vibrio cholerae* (*Vibrionaceae*), increased the transcription of the putative DNA uptake genes and induced weak DNA uptake in *E. coli* [16], [17]. But neither any of these putative DNA uptake gene orthologs nor *sxy* was found to mediate natural transformation of *E. coli* on plates [18]. The kinetics of transformation as a function of DNA concentration suggests the presence of a different route for the entry of double stranded DNA during natural transformation of *E. coli* [18]. This new type of DNA transfer relies on agar in plates but is unrelated to the divalent cations (e.g. Ca^2+^, Mg^2+^, Mn^2+^) contained in the agar. Further analysis showed that natural transformation of *E. coli* on plates was regulated by an alternative sigma factor RpoS [13]. However, it remains mysterious how DNA transfers across bacterial membranes and how RpoS regulates DNA transfer on the solid surface.

OmpA, one of the most abundant outer membrane proteins (OMPs) expressed highly at the exponential growth phase but moderately at the stationary phase in *E. coli*, is involved in accepting the conjugative plasmid DNA and the bacteriophage DNA, both of which are coated with proteins [19]–[25], as well as in the transport of bacteriocins, a group of toxic peptides and proteins [26]–[28]. But it remains unknown whether OmpA participates in DNA transfer during transformation of *E. coli*. In this study, we made attempts to explore potential roles of OmpA in mediating DNA transfer during natural and artificial transformation of *E. coli* on agar plates and in solution respectively.

## Results

### 1. Transformation is Promoted by *ompA* Inactivation on Agar Plates

**Figure 1:**
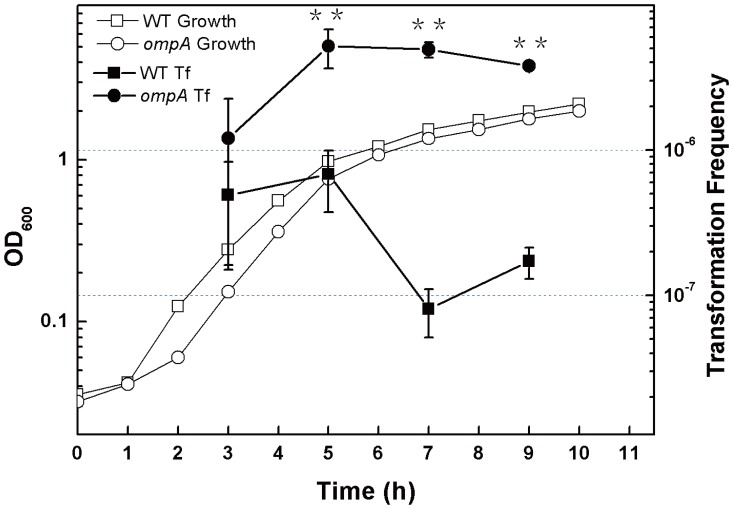
Effect of *ompA* inactivation on natural transformation on 5% agar plates. Overnight grown (13 hr) *E. coli* BW25113 (square symbols) and JW0940 (BW25113::*ompA*::kan, circle symbols) were inoculated (1%) in 100 ml of fresh LB. While the cultures were incubated in a shaker with a speed of 150 rpm at 30°C, the optical density at OD_600_ was measured periodically (open symbols). Transformation was performed as described in Materials and Methods. The mixture of the cell culture and pDsRED was plated on selective plates with 5% agar. Viable counts were measured by plating diluted culture on the same plates without any antibiotics. Transformation frequency (filled symbols) was calculated by dividing the number of transformants per ml by the number of viable counts per ml. Each point denotes an average of 4 samples. Error bars denote standard deviation. ^*^ P value ≦ 0.05; ^**^ P value ≦ 0.01.

The finding that orthologs of DNA uptake genes do not mediate natural transformation in *E. coli* suggests the presence of new routes for DNA transfer. The multiple roles of OmpA in DNA transfer during conjugation and bacteriophage infection, as well as colicin transport, stimulated us to test its potential role in natural transformation on agar plates. An *ompA* mutant JW0940 was obtained from the Keio collection [29] and its genotype was examined. PCR analysis showed that the *ompA* gene was replaced by a kanamycin resistance gene as predicted (Fig. S1). To check whether *ompA* is involved in transformation on agar plates, we compared transformation frequencies in JW0940 and its wild type parent BW25113. JW0940 and BW25113 showed a similar growth pattern, but their transformation frequencies varied largely (Fig. 1). After 5 to 9 hours of incubation, transformation frequencies of JW0940 were ∼5×10^−6^, which was 7 to 60 folds higher than those of BW25113 (Fig. 1). This result shows that the inactivation of *ompA* significantly improves transformation of *E. coli* on agar plates, indicating that OmpA blocks the transfer of naked plasmid DNA.

### 2. *ompA* Inactivation Promotes Transformation on Plates with a Low Concentration of Agar

**Figure 2:**
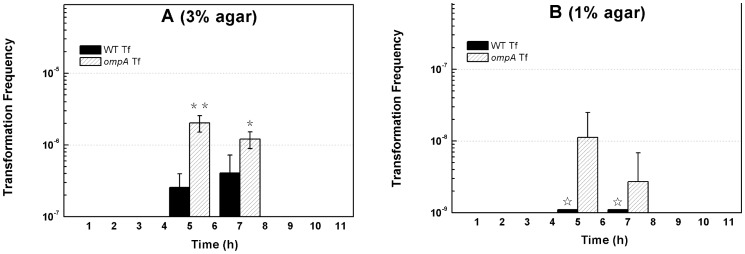
Effect of *ompA* inactivation on natural transformation on soft plates containing less agar. OD_600_ (open symbols) of *E. coli* BW25113 (square symbols) and JW0940 (BW25113::*ompA*::kan, circle symbols) were measured periodically. Transformation of *E. coli* BW25113 (solid column) and JW0940 (open column) was performed as described in Fig. 1 except for the concentration of agar in plates. (A) Transformation on plates with 3% agar. (B) Transformation on plates with 1% agar. Each column denotes an average of 4 samples. Error bars denote standard deviation. ^*^ P value ≦ 0.05; ^**^ P value ≦ 0.01; ^☆^ transformation frequency <10^−9^.

During transformation of *E. coli* on plates, OMPs should directly contact with the surface of agar plates where DNA uptake occurs [13]. Our previous work demonstrated that agar significantly promotes transformation on plates [18]. We considered whether a solid surface was important to DNA transfer suppression by OmpA on agar plates. The hardness of the surface of plates is determined by the concentration of agar. We examined the effect of *ompA* inactivation on transformation with plates containing agar at lower concentrations. On the plates with 3% agar, we observed that transformation frequency was 1∼2 × 10^−6^ in JW0940 (*ompA*–) after 5∼7 h incubation, significantly higher than that in its wild-type parent BW25113 (2∼4×10^−7^) at the same growth stage (Fig. 2A). On the soft plates with 1% agar, we failed to detect any transformants in BW25113 (detection limit: 1×10^−9^) (Fig. 2B), while weak but detectable transformation was observed with JW0940 as the recipient cell. After 5∼7 hours of bacterial growth, transformation frequency of JW0940 was 10^−9^∼10^−8^ on plates containing 1% agar (Fig. 2B). These data clearly show that OmpA still blocks the transfer of plasmid DNA on the softer plates, indicating that the effect of OmpA on DNA transfer does not require a surface with high hardness during natural transformation of *E. coli* on plates.

### 3. Chemical Transformation is Reduced by *ompA* Inactivation in Solution

**Figure 3:**
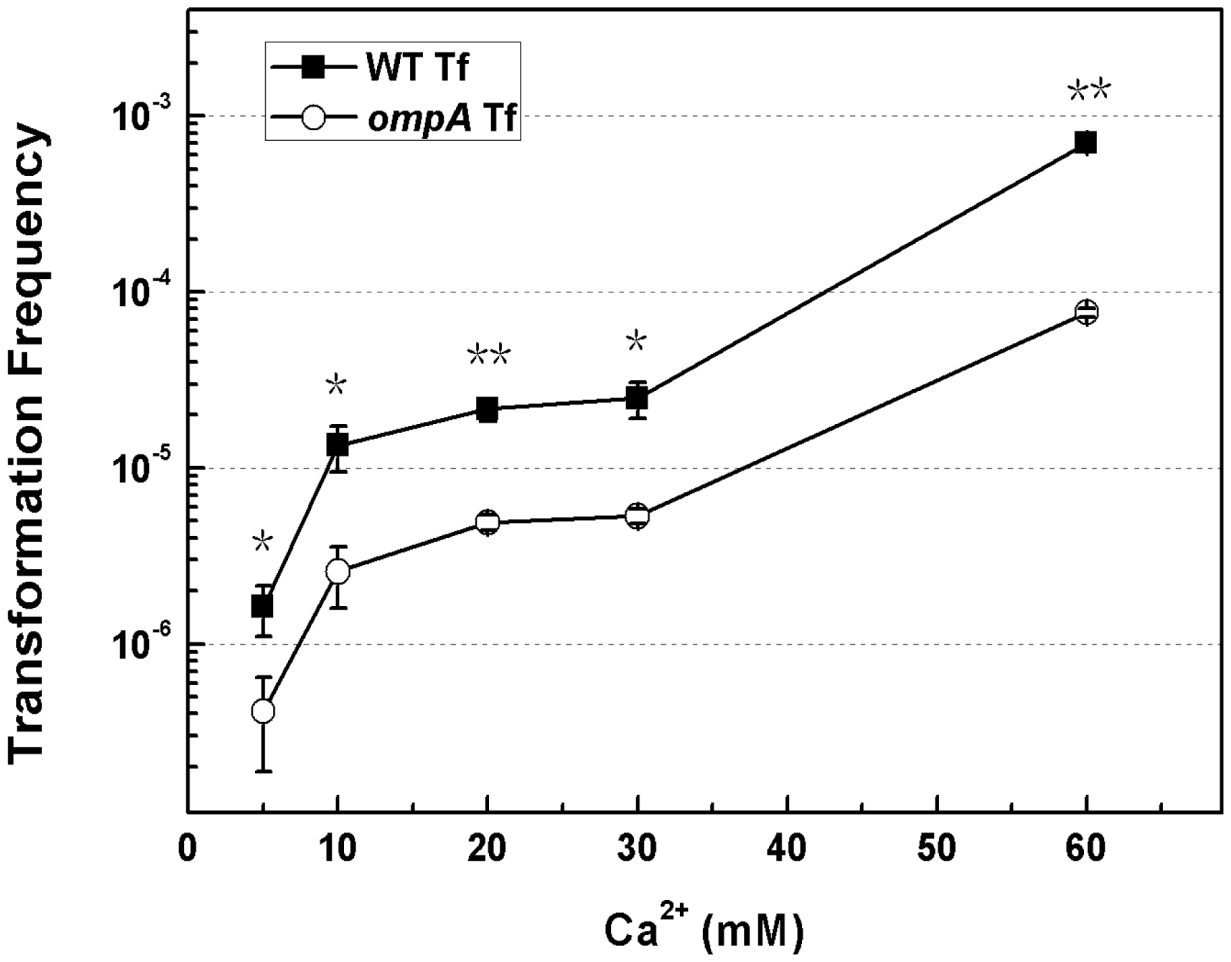
Effect of *ompA* inactivation on chemical transformation with a series of concentrations of Ca^2+^. Competent *E. coli* cells were prepared with CaCl_2_ solution at serial concentrations (5, 10, 20, 30 and 60 mM). Transformation frequencies of BW25113 (solid square symbols) and the *ompA* mutant JW0940 (BW25113::*ompA*::kan, open circle symbols) were shown. Each point denotes an average of 2 samples. Error bars denote standard deviation. ^*^P value ≦ 0.05; ^**^P value ≦ 0.01.

After knowing that OmpA inhibited DNA transfer on agar plates while the hardness of the surface of the plate did not affect the suppression effect of OmpA on transformation, we considered whether OmpA affected DNA transfer during chemical transformation of *E. coli* in liquid culture. Chemical competent *E. coli* BW25113 and JW0940 (*ompA*–) cells were prepared with 60 mM of CaCl_2_ and transformed with the plasmid pDsRED. We found that chemical transformation frequencies of the *ompA* mutant JW0940 were systematically lower than its wildtype in repeated experiments (n >8). In a typical experiment, the chemical transformation frequency of JW0940 was 7.63 × 10^−5^, about 10-fold lower than its wild type parent BW25113 (Fig. 3). The result demonstrates that the inactivation of *ompA* reduces chemical transformation frequency, suggesting that OmpA facilitates DNA transfer in the liquid CaCl_2_ solution.

### 4. *ompA* Inactivation Still Reduces Chemical Transformation in the Liquid with a Low Concentration of Ca^2+^

In the above experiments, we observed that OmpA played opposite roles in natural and chemical transformation: it promotes DNA transfer in the former but reduces DNA transfer in the latter. A high concentration of Ca^2+^ in liquid is essential to chemical transformation while the addition of Ca^2+^ was not necessary for natural transformation on agar plates [7], [18]. To examine whether Ca^2+^ played an important role in functional reversal of OmpA in mediating DNA transfer during natural and chemical transformation, we compared chemical transformation frequencies of BW25113 (wildtype) and JW0940 (*ompA*–) in the Ca^2+^ solution at a series of low concentrations. When no CaCl_2_ was added, no transformants were detected in both BW25113 and JW0940. With the increase of Ca^2+^ concentration from 5 mM to 30 mM, transformation frequencies of both BW25113 and JW0940 increased; while the transformation frequency of JW0940 was consistently significantly lower than BW25113 in solution with Ca^2+^ at any concentrations tested (Fig. 3). These data demonstrate that the promotion effect of OmpA on chemical transformation does not rely on a high concentration of Ca^2+^, suggesting that the increase of Ca^2+^ is not sufficient to reverse the function of OmpA in mediating DNA transfer.

### 5. Heat Shock does not Affect the Reduction of Chemical Transformation by *ompA* Inactivation

**Figure 4:**
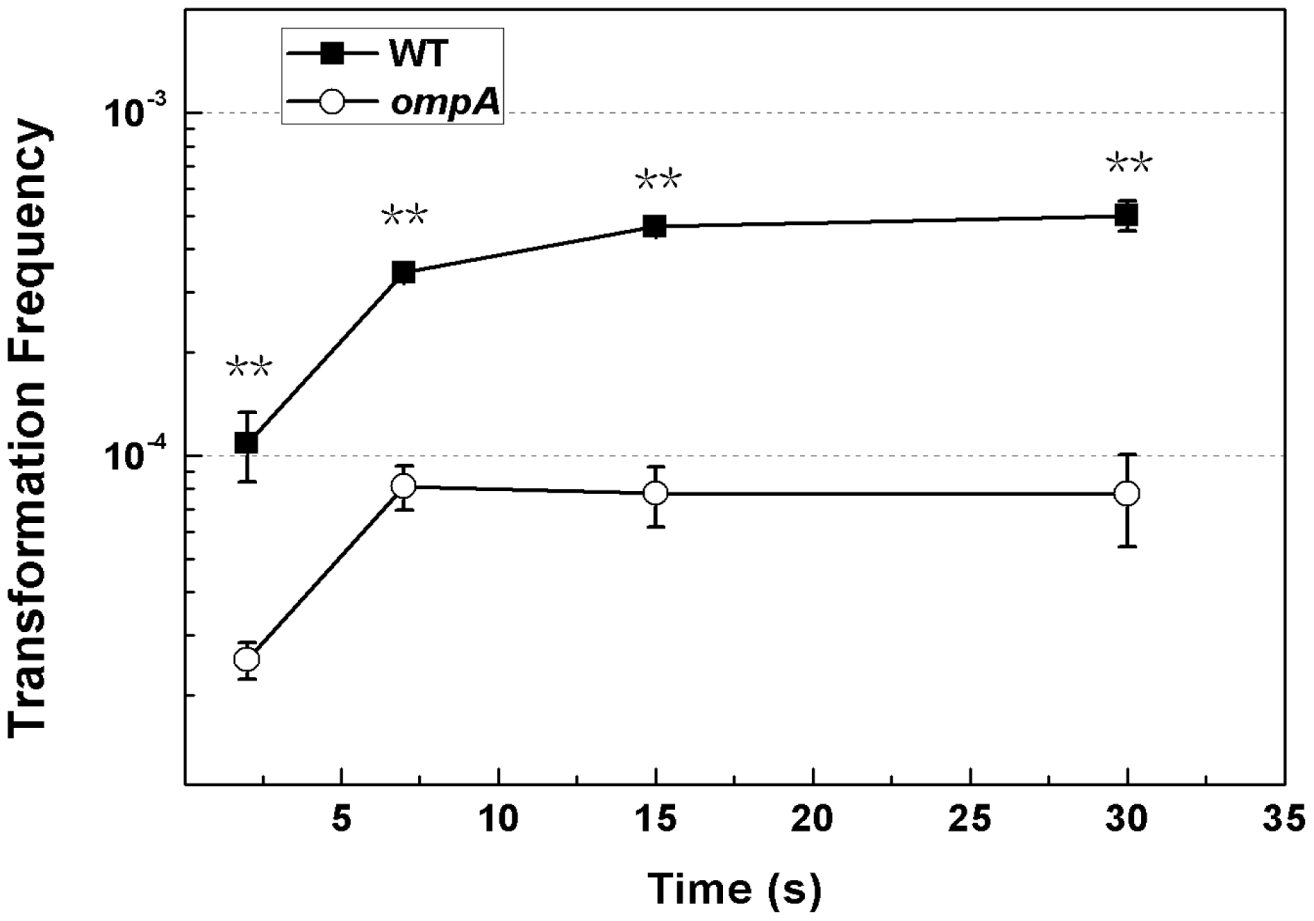
Effect of *ompA* inactivation on chemical transformation with a shortened time for heat shock. Competent *E. coli* cells were prepared with CaCl_2_ solution at 60 mM. The mixture of competent cells and pDsRED on ice was placed in the water bath (42°C) for 2∼30 seconds. Chemical transformation frequencies of *E. coli* BW25113 (solid square symbols) and JW0940 (BW25113::*ompA*::kan, open circle symbols) were shown. Each point denotes an average of 2 to 4 samples. Error bars denote standard deviation. ^*^P value ≦ 0.05; ^**^P value ≦ 0.01.

It has been reported that the increase of temperature facilitates the transition of the narrow OmpA pore to the large pore, whose conformation is thermally irreversible [30], [31]. To check whether the temperature shift from 0 to 42°C caused the reversal of OmpA from the DNA blocker to the booster, we compared chemical transformation frequencies in the *ompA* mutant JW0940 and the wildtype BW25113 in chemical transformation with a shortened time for heat shock. The transformation frequency of BW25113 was still consistently significantly higher than JW0940 in chemical transformation with 2, 7, 15 or 30 seconds for heat shock (Fig. 4). This result indicates that the heat shock should not be the main mechanism for functional reversal of OmpA in mediating DNA transfer.

### 6. *ompA* is not Regulated by RpoS

**Figure 5:**
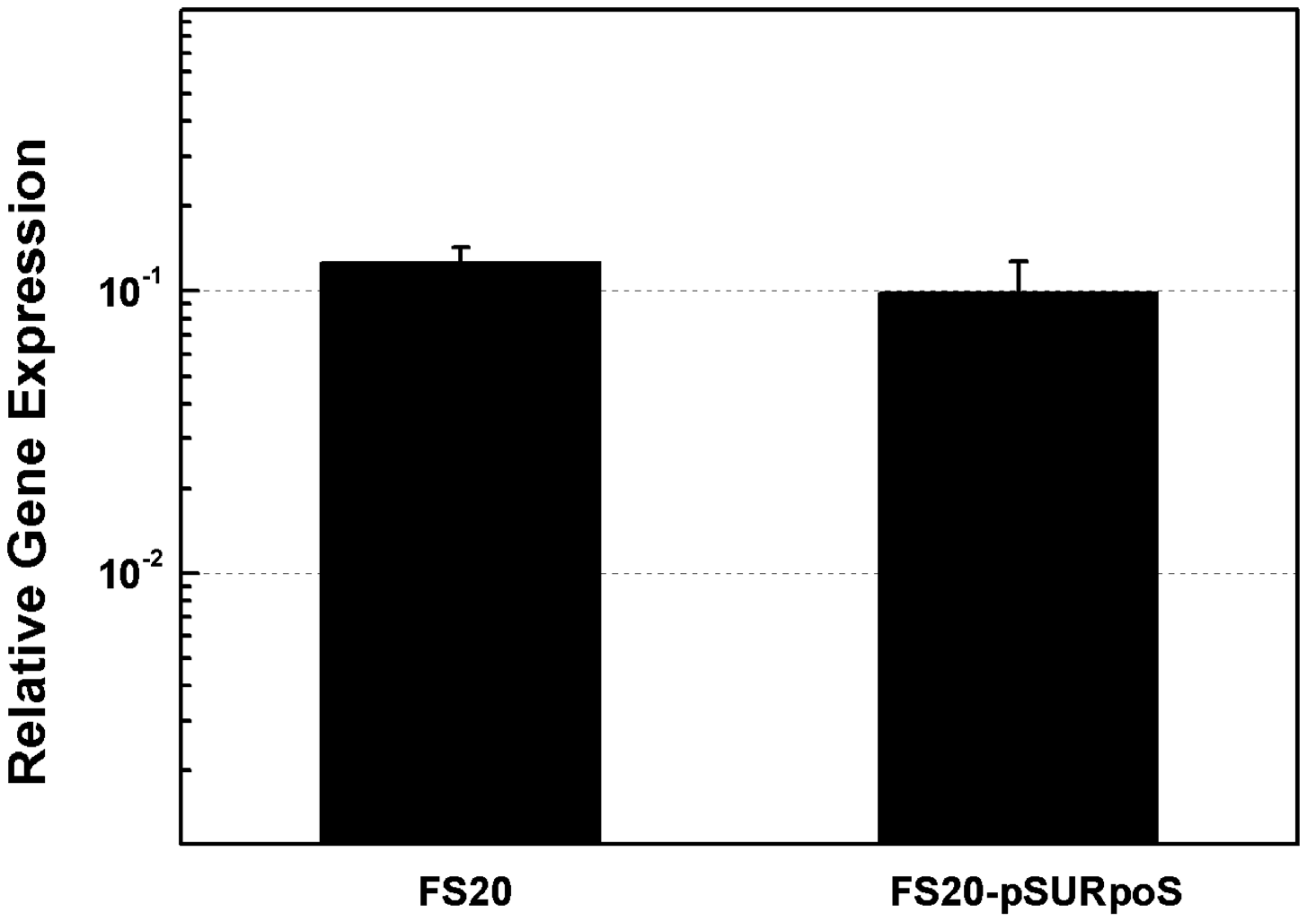
Quantification of *ompA* transcripts in the *rpoS*– and the *rpoS*+ strains. The transcription of *ompA* was quantified in strains FS20-pSU (*rpoS–*) and FS20-pSURpoS (*rpoS*+) with Real-time PCR using 16s rRNA as the reference. Each column denotes an average of 4 samples. Error bars denote standard deviation.

The stationary phase master regulator RpoS affects the transcription of more than 10% of *E. coli* genes [32]. Our previous work showed that natural transformation of *E. coli* is regulated by the transcription regulator RpoS [13], which is able to compete with σ^70^ [33], the principle regulator of *ompA* (Fig. S2). To check whether RpoS regulates transformation by suppressing *ompA* transcription, we compared the transcription of *ompA* in the *rpoS*– and the *rpoS*+ strains through quantitative PCR (qPCR). The amount of *ompA* transcripts did not show a significant difference in strains FS20-pSU (*rpoS*–) and FS20-pSURpoS (*rpoS*+) (Fig. 5), demonstrating that the transcription of *ompA* is not affected by RpoS. Therefore, the effect of OmpA on DNA transfer should be unrelated to RpoS which may target on other DNA uptake or processing genes for transformation on agar plates. It has been reported that the amount of OmpA decreases as a result of the reduction of the *ompA* transcripts by the small RNA MicA, which facilitates ribonuclelytic degradation [34]. Considering that OmpA blocks DNA transfer in transformation, we suppose that the expression of *ompA* at a low level could be a reason for the relative high transformation frequency at the stationary phase.

## Discussion

In this study, we showed that OmpA played opposite roles in mediating DNA transfer during natural and chemical transformation: it blocks DNA transfer during natural transformation (Fig. 1) but boosts DNA transfer in chemical transformation of *E. coli* (Fig. 3). While the suppression effect of OmpA on DNA transfer is not limited by the concentration of agar in natural transformation on plates (Fig. 2) and the promotion effect of OmpA on DNA transfer is unaffected by the two pivotal factors (Ca^2+^ and heat shock), which are essential to chemical transformation (Fig. 3, 4). To explain opposite roles of OmpA in DNA transfer, we reasoned that a single factor or several associated factors might determine its functions. Because neither changing agar concentration in natural transformation nor reducing Ca^2+^ or shortening temperature shift time in chemical transformation affected the function of OmpA in chemical transformation, we conclude that solely agar or Ca^2+^ or heat shock can not determine the role of OmpA in DNA transfer. We inclined to propose that Ca^2+^ and heat shock, possibly together with other factors, may cooperate in facilitating OmpA mediated chemical transformation. Nevertheless, our data do not exclude the possibility that a single unidentified factor can reverse the function of OmpA in DNA transfer. In addition, we have shown that OmpA is not a target of the DNA transfer regulator RpoS in natural transformation of *E. coli* (Fig. 5).

Structure analysis shows that the OMP OmpA contains extracellular loops, an eight-stranded transmembrane β-barrel embedded in the outer membrane and a C-terminus in the periplasm where it associates with the peptidoglycan [31], [35]–[37]. It forms a gated pore which is permeable to ions and larger solutes [35], [38]–[40]. In both bacteriophage infection and conjugation, it is commonly thought that OmpA affects the transfer of DNA by interacting with related proteins [19]–[25]. For example, OmpA functionally interacts with the F plasmid-encoded OMP TraN in conjugation and DNA transfer was proposed to be mediated by the OMP-to-OMP interaction [23], [25]. In this study, we discovered that OmpA blocked the transfer of non-protein-coated plasmid DNA in natural transformation of *E. coli* on agar plates, but promoted the transfer of naked DNA in chemical transformation (Fig. 1, 3). Considering that OmpA locates at the outer membrane, it should act on DNA transfer by limiting/promoting its entry. Inactivation of *ompA* increased transformation on agar plates by 7 to 60 folds (Fig. 1). Accordingly, OmpA should prevent the entry of more than 86%∼98% of transforming DNA on the solid surface during natural transformation. While in solution, inactivation of *ompA* reduced transformation by 4 to 10 folds (Fig. 2), suggesting that OmpA may accommodate the entry of 75%∼90% transforming DNA in chemical transformation; the remaining DNA may pass across the bacterial membrane through another channel formed by poly-beta-hydroxybutyrate/calcium polyphosphate [41]–[44]. To our knowledge, this is the first report demonstrating that OmpA plays opposite roles in the transfer of DNA in bacterial transformation.

In the normal conditions, OmpA is unlikely to form an open gate [35]–[37], but it can be switched to the ‘gate-open’ state under the drive of molecular forces [45]. The open and close of the gate was reported to depend on a salt bridge formed by Arg138-Glu52 interaction [45]. To reconcile the contrary roles of OmpA in DNA transfer in natural and artificial transformation systems, we provide an explanation as follows: naked DNA might be trapped in OmpA at the default ‘gate-closed’ state and therefore be unable to contact with the right channel for its entry during natural transformation on the surface of agar plates (see Fig. S3A). Artificial treatments may help open the gate to allow DNA to pass across the channel formed by OmpA in liquid during chemical transformation (see Fig. S3B). The diameter of double stranded DNA is approximate 2.4 nm. If DNA indeed passed across the outer membrane through the OmpA channel in chemical transformation, the size of the pore formed by OmpA could be as large as 2.4 nm, larger than the predicted size of ∼1 nm, inferred by the dependence of swelling rates on the size of permeable solutes [39], [46].

Our previous work showed that agar stimulates transformation on plates and that the effect of agar on transformation stimulation can be suppressed by EGTA which chelates divalent cations [18]. Therefore, agar could affect DNA transfer by either providing unknown cation(s) which may affect the activity of proteins or increasing the hardness of the surface of the plate which may contact with the OMPs directly. If the effect of OmpA on natural transformation depends on the cation(s) or hardness of the surface provided by a high concentration of agar on plates, OmpA would be unable to block DNA transfer on soft plates which contain less agar. However, our experiments showed that the inactivation of *ompA* still improved transformation on soft plates containing 1% or 3% agar (Fig. 3), indicating that the effect of OmpA on natural transformation does not depend on the cation(s) or hardness of the surface provided by a high concentration of agar on plates. Sinha and Redfield reported that although over-expression of the competence regulator Sxy induced the expression of DNA uptake genes, only a little DNA was taken up into *E. coli* cells in the liquid culture [17]. Considering our observation that OmpA suppresses DNA transfer independent of agar, it is probably that OmpA could restrict the entry of naked DNA and result in poor DNA uptake in the liquid in their studies.

## Materials and Methods

### 1. Natural Transformation of *E. coli* on Agar Plates

All of the strains, plasmids and primers used in the present study are listed in Table 1. Transformation was carried out by using a procedure that was previously described [13]. All experiments were performed at a room temperature (30°C). To prepare competent cells, 1 ml of the overnight grown pre-culture was inoculated into 100 ml of 1.5 × LB broth in a triangle glass flask. Cell growth was measured by recording the optical cell density at 600 nm (OD_600_). At intervals, 1 ml of the culture was precipitated and 900 µl of the supernatant was discarded. The cell pellet was resuspended in the remaining 100 µl of the supernatant and 4 µg of plasmid pDsRed (final concentration, 40 µg/ml) was added. For each sample, 50 µl of the above mixture was plated onto LB plates containing 5% agar (or 1% agar or 3% agar when required) and 200 µg/ml of ampicillin, which had been air dried at room temperature (30°C) for 24∼48 hours. Transformation frequency was calculated by dividing the number of transformants by viable counts.

**Table 1:** Strains, plasmids and primers used in this study.

*E. coli* strain, plasmids, or primers	Relevant genotype and/or description	Source or reference
**Strains**		
BW25113	F- λ- Δ*(araD-araB)567* Δ*lacZ4787*(::*rrnB*-3) *rph-1 hsdR514* Δ*(rhaD-rhaB)568*	[29]
JW0940	BW25113::*ompA*::kan^R^	[29]
FS20-pSU	MC4100::*rpoS*:: Kan^R^; containing pSU, Cm^R^	[13]
FS20-pSURpoS	MC4100::*rpoS*:: Kan^R^; containing pSURpoS, Cm^R^	[13]
**Plasmid**		
pDsRED	pUC replicon; red-fluorescence-protein (RFP) expressing plasmid; Amp^r^	[12]
**Primers**		
*ompA*(CHK) Forward	cgaagatatcggtagagtt	This study
*ompA*(CHK) Reverse	cgctttctgaaacgattgt	This study
*ompA* (qPCR) Forward	cttcgctggcggtgttgag	This study
*ompA* (qPCR) Reverse	acgagtgccgatggtgtgt	This study
16S rDNA (qPCR) Forward	tgcatctgatactggcaagc	[13]
16S rDNA (qPCR) Reverse	acctgagcgtcagtcttcgt	[13]

### 2. RNA Isolation and Real-time Quantitative PCR

RNA from the FS20-pSU (carrying *rpoS* null mutation), FS20-pSURpoS (RpoS over-expressing strain) was isolated and reverse transcribed to cDNA with TransScript Reverse Transcriptase (TransGen Biotech). Duplicate PCRs were run for each cDNA sample with a method that was previously described using 16 s rRNA as the reference gene [13]. The threshold cycles were calculated with the Bio-Rad CFX-96 manager software and the relative expression of genes was calculated by the formula 2^(ΔCt Taget − ΔCt Reference)^.

### 3. Chemical Transformation of *E. coli*

When the *E. coli* culture was grown to an OD_600_ of ∼0.5, competent cells were prepared by a documented method with slight modifications [7]. Briefly, exponentially growing *E. coli* cells were harvested at 4°C, washed with 60 mM CaCl_2_ solution and resuspended in the CaCl_2_ solution on ice. Plasmid pDsRED (final concentration, 20 µg/ml) was added to the competent cells, which were placed on ice for 30 minutes before a heat shock at 42°C for 90 seconds. To check the effect of Ca^2+^ on chemical transformation, competent cells were prepared with the CaCl_2_ solution at concentrations of 5, 10, 20 and 30 mM. To check the effect of heat shock on chemical transformation, the mixture of competent cells and DNA on ice was transferred to the 42°C water bath for 2, 7, 15 and 30 seconds. Transformation frequency was calculated by dividing the number of transformants by viable counts.

## Supporting Information

Figure S1 Examination of the construction the *ompA* mutant. PCR experiments confirmed the loss of wild-type gene fragments in the *ompA* mutant strains from Keio collection and its replacement by a fragment. The sizes of the fragments from the *ompA* mutant and its wildtype parent were fully consistent with that predicted from simple insertion of kanamycin-resistance gene cassette. (A) Analysis of the sizes of PCR fragments for confirmation of the structure of the *ompA* mutant and its wild type. (B) Genetic organization of *ompA* in chromosome and location of cassette insertion. Sequences of the primers for examination were listed in Table 1 [*ompA* (CHK) forward/reverse].
(TIF)

Figure S2 Regulation of *ompA* expression (EcoCyc website: http://www.ecocyc.org/). The transcription of *ompA* is driven by σ^70^. The Hfq dependent small RNA MicA is able to bind with the RBS site of the *ompA* mRNA and mediates its destabilization by RNase E.
(TIF)

Figure S3 Proposed explanation for opposite roles of OmpA in DNA transfer. (A) ‘Gate-closed’ state: the formation of the salt bridge Arg138-Glu52 occludes ions in the β-barrel of OmpA. In natural transformation, OmpA may be under the ‘gate-closed’ state and prevent the entry of DNA. (B) ‘Gate-open’ state: the break of Arg138-Glu52 salt bridge opens the gate and makes OmpA permeable to ions. In chemical transformation, artificial treatments may help break the Arg138-Glu52 salt bridge and allow the entry of DNA.
(TIF)
