# Supplementary figures and images for: Block and Boost DNA Transfer: Opposite Roles of OmpA in Natural and Artificial Transformation of Escherichia coli
Source: PLoS One. 2013 Mar 22;8(3):e59019. doi: 10.1371/journal.pone.0059019 (PMC3606455; doi:10.1371/journal.pone.0059019)

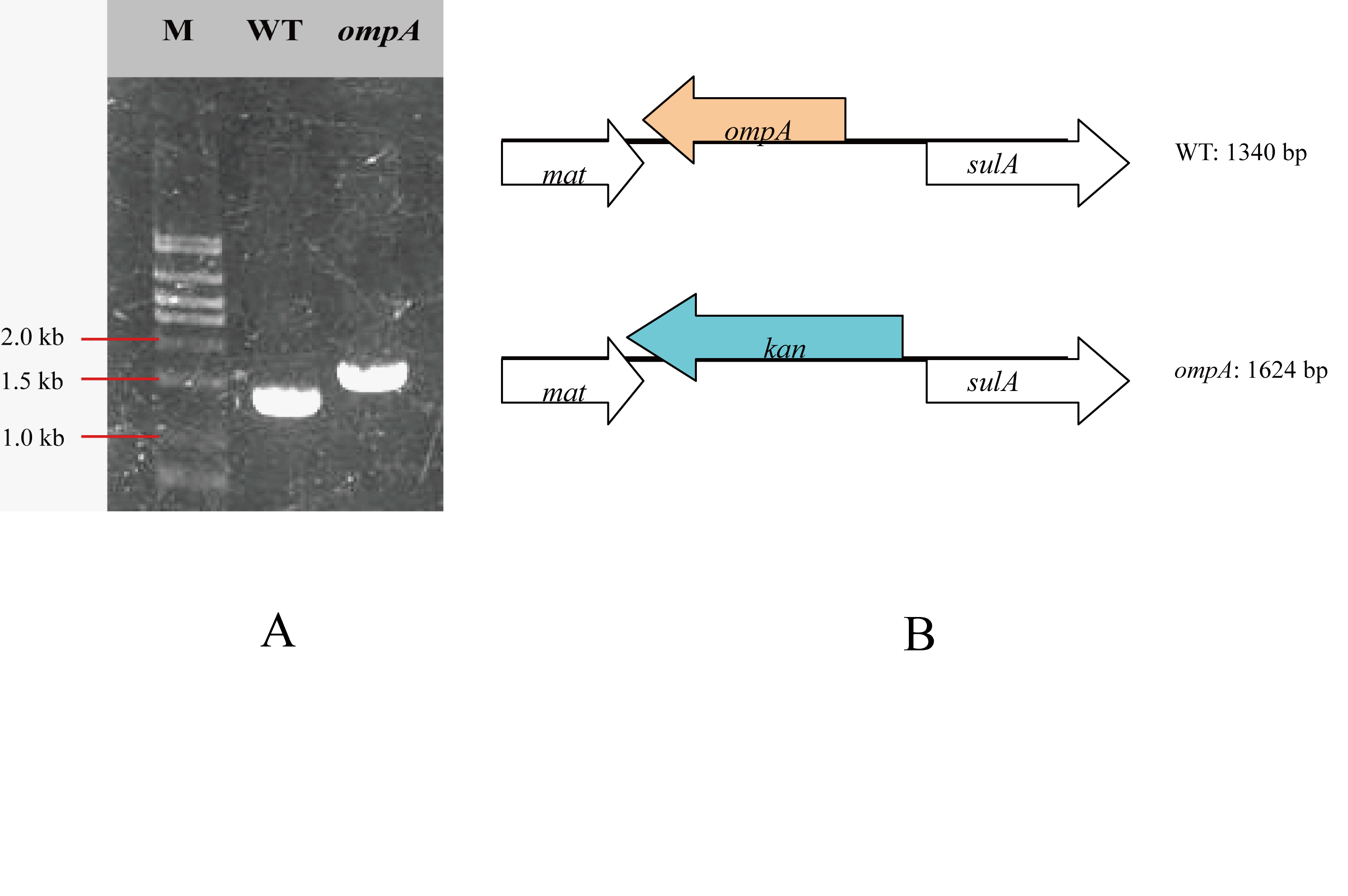

Supplement: Figure S1 — Examination of the construction the ompA mutant. PCR experiments confirmed the loss of wild-type gene fragments in the ompA mutant strains from Keio collection and its replacement by a fragment. The sizes of the fragments from the ompA mutant and its wildtype parent were fully consistent with that predicted from simple insertion of kanamycin-resistance gene cassette. (A) Analysis of the sizes of PCR fragments for confirmation of the structure of the ompA mutant and its wild type. (B) Genetic organization of ompA in chromosome and location of cassette insertion. Sequences of the primers for examination were listed in Table 1 [ompA (CHK) forward/reverse]. (TIF) [file pone.0059019.s001.tif]

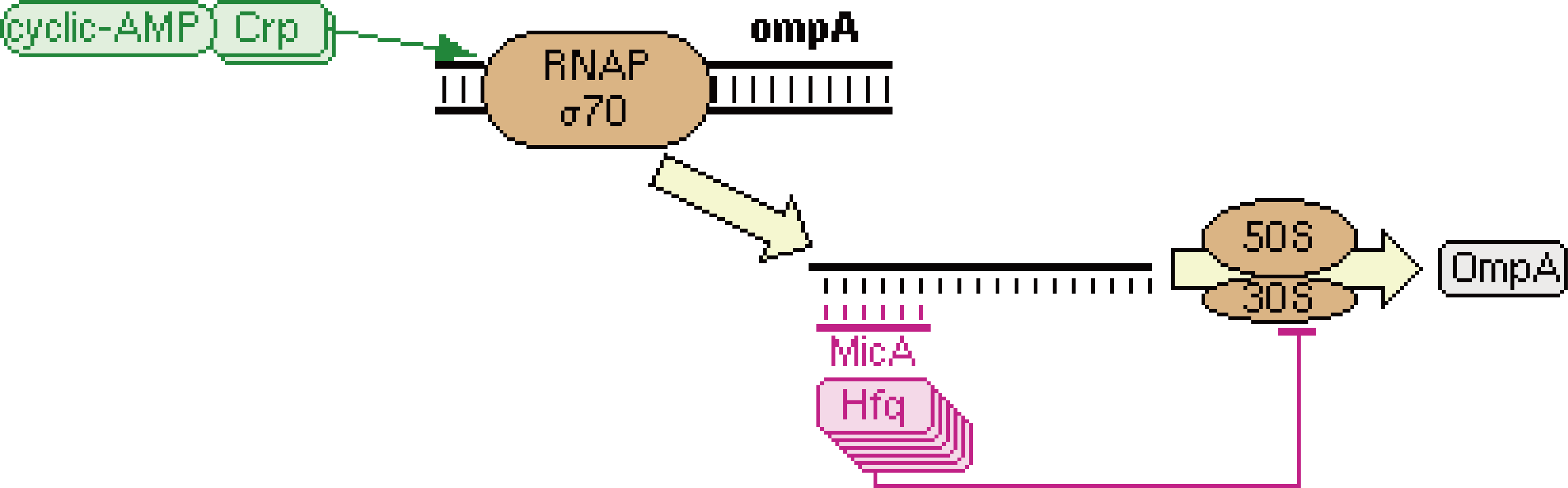

Supplement: Figure S2 — Regulation of ompA expression (EcoCyc website: http://www.ecocyc.org/). The transcription of ompA is driven by σ70. The Hfq dependent small RNA MicA is able to bind with the RBS site of the ompA mRNA and mediates its destabilization by RNase E. (TIF) [file pone.0059019.s002.tif]

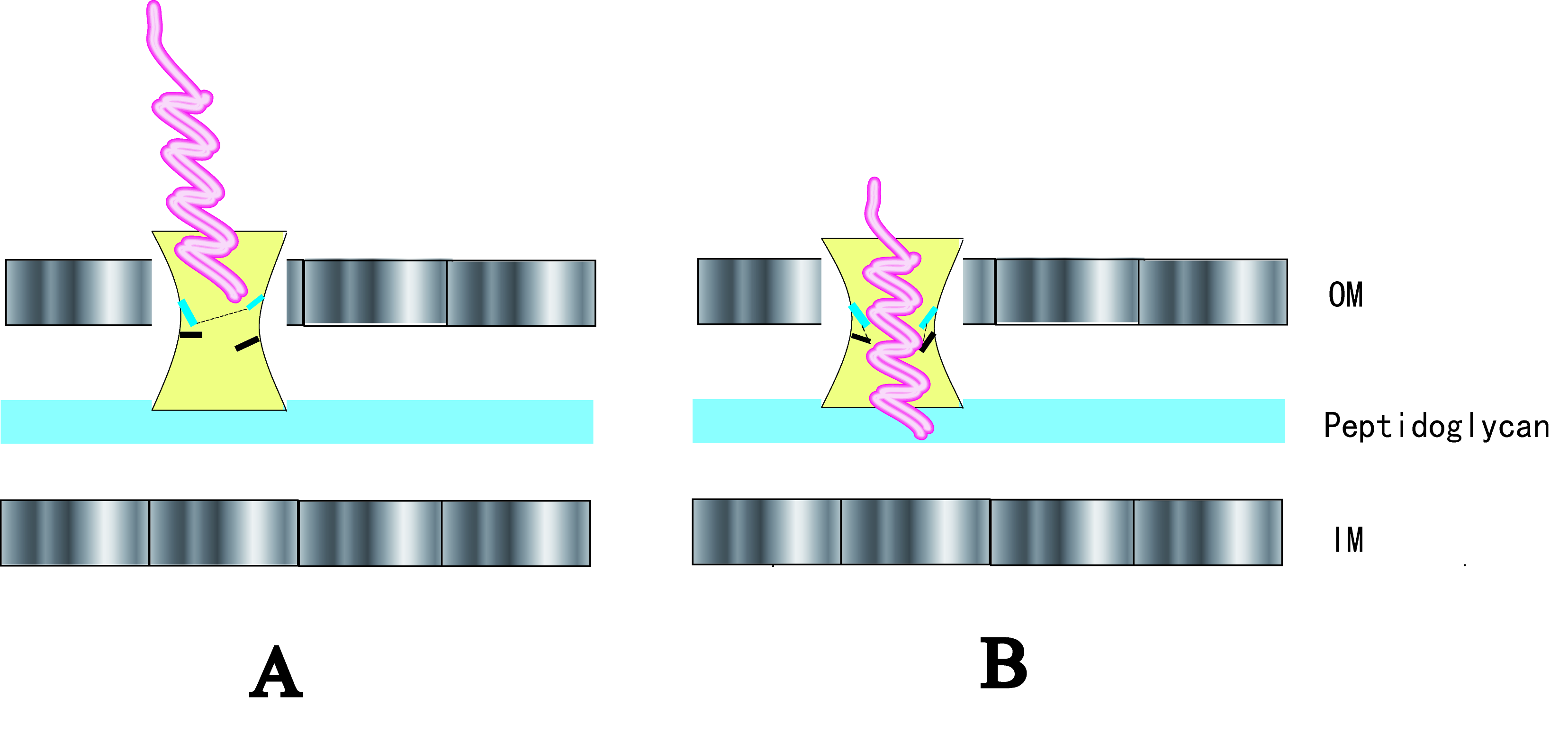

Supplement: Figure S3 — Proposed explanation for opposite roles of OmpA in DNA transfer. (A) ‘Gate-closed’ state: the formation of the salt bridge Arg138-Glu52 occludes ions in the β-barrel of OmpA. In natural transformation, OmpA may be under the ‘gate-closed’ state and prevent the entry of DNA. (B) ‘Gate-open’ state: the break of Arg138-Glu52 salt bridge opens the gate and makes OmpA permeable to ions. In chemical transformation, artificial treatments may help break the Arg138-Glu52 salt bridge and allow the entry of DNA. (TIF) [file pone.0059019.s003.tif]
